# Examining anterior prefrontal cortex resting-state functional connectivity patterns associated with depressive symptoms in chronic moderate-to-severe traumatic brain injury

**DOI:** 10.3389/fneur.2025.1541520

**Published:** 2025-03-28

**Authors:** Layan A. Elfaki, Bhanu Sharma, Liesel-Ann C. Meusel, Isis So, Brenda Colella, Anne L. Wheeler, Jocelyn E. Harris, Robin E. A. Green

**Affiliations:** ^1^Temerty Faculty of Medicine, University of Toronto, Toronto, ON, Canada; ^2^The KITE Research Institute, Toronto Rehabilitation Institute, University Health Network, Toronto, ON, Canada; ^3^Department of Electrical and Computer Engineering, McMaster University, Hamilton, ON, Canada; ^4^Schulich School of Medicine and Dentistry, Western University, London, ON, Canada; ^5^Neuroscience and Mental Health Program, The Hospital for Sick Children, Toronto, ON, Canada; ^6^Physiology Department, University of Toronto, Toronto, ON, Canada; ^7^Faculty of Health Sciences, School of Rehabilitation Science, McMaster University, Hamilton, ON, Canada; ^8^Rehabilitation Sciences Institute, Temerty Faculty of Medicine, University of Toronto, Toronto, ON, Canada

**Keywords:** chronic moderate-to-severe traumatic brain injury, depression, anterior prefrontal cortex, seed-based connectivity analysis, personality assessment inventory, fusiform gyrus

## Abstract

In chronic moderate-to-severe TBI (msTBI), depression is one of the most common psychiatric consequences. Yet to date, there is limited understanding of its neural underpinnings. This study aimed to better understand this gap by examining seed-to-voxel connectivity in depression, with all voxel-wise associations seeded to the bilateral anterior prefrontal cortices (aPFC). In a secondary analysis of 32 patients with chronic msTBI and 17 age-matched controls acquired from the *Toronto Rehab TBI Recovery Study* database, the Personality Assessment Inventory Depression scale scores were used to group patients into an *msTBI-Dep group* (*T* ≥ 60; *n* = 13) and an *msTBI-Non-Dep group* (*T* < 60; *n* = 19). Resting-state fMRI scans were analyzed using seed-based connectivity analyses. *F*-tests, controlling for age and education, were used to assess differences in bilateral aPFC rsFC across the 3 groups. After nonparametric permutation testing, the left aPFC demonstrated significantly increased rsFC with the left (*p* = 0.041) and right (*p* = 0.013) fusiform gyri, the right superior temporal lobe (*p* = 0.032), and the right precentral gyrus (*p* = 0.042) in the *msTBI-Dep group* compared to controls. The *msTBI-Non-Dep group* had no significant rsFC differences with either group. To our knowledge, this study is the first to examine aPFC rsFC in a sample of patients with msTBI exclusively. Our preliminary findings suggest a role for the aPFC in the pathophysiology of depressive symptoms in patients with chronic msTBI. Increased aPFC-sensory/motor rsFC could be associated with vulnerability to depression post-TBI, a hypothesis that warrants further investigation.

## 1 Introduction

Depression is one of the most frequently reported psychiatric sequelae of moderate-to-severe traumatic brain injury (msTBI), affecting 26–40% of survivors (1–3). A substantial portion of patients develop depressive symptoms in the chronic stages (operationalized here as >6 months) post-injury (4–7), which can impede ongoing cognitive and functional recovery and is associated with increased suicidal ideation (2, 8–10). A better understanding of the neural basis of depression in chronic msTBI informs treatment research through offering an objective and quantitative biomarker as a treatment target/outcome measure that can supplement behavioral outcome measures. As well, such a biomarker can be used for neuromodulation research e.g., transcranial direct current stimulation (11–13).

Resting-state functional connectivity (rsFC) is a correlation of the blood oxygen level-dependent timeseries of two voxels or regions of interest and may constitute a data-driven avenue to explore depression in msTBI (14, 15). rsFC patterns have been extensively studied in patients with major depressive disorder through non-invasive methods (16–18) and are reproducible across various task states (19, 20). With regard to the neuroimaging correlates of depression following TBI of all severities, a recent systematic review identified 10 rsFC studies on comorbid depression and TBI (21), although only three studies examined patients with a moderate-to-severe injury not exclusively (22–24). Irrespective of TBI severity, notably inconsistent results were reported across the 10 studies, potentially attributable to their variable data acquisition and neuroimaging processing methods, choice of regions of interest, and differing clinical and demographic sample characteristics. Further, different studies operationalize depression using different cutoffs, depressive phenotypes, and time post-injury. These discrepancies may explain the limited progress in identifying a biomarker of depression in TBI (14, 21, 25). This is similarly the case for non-TBI related depression despite it being extensively studied (26–29).

The anterior prefrontal cortex (aPFC; Brodmann area 10 (BA10)) is a relevant target given the documented occurrence of cognitive control deficits in both depression (30–32) and msTBI (33–35) independently. It is implicated in cognitive control as a hub within the frontoparietal control network (36, 37) as well as salience detection as part of the salience network (38–40). To our knowledge, only two studies have investigated aPFC rsFC in patients with comorbid depression and chronic TBI (22, 23). The first utilized seed-based connectivity analyses and reported reduced rsFC connectivity between the aPFC and regions including the superior temporal gyrus, frontal pole, and the inferior parietal lobule in patients with TBI, irrespective of depressive symptom severity, as compared to healthy controls (22). In a subsequent study by this group using an expanded participant cohort, the authors examined the effects of cognitive training on depressive symptoms in patients with chronic TBI and depressive symptoms (23). Results revealed that training-induced reductions in cognitive depressive symptom severity were positively associated with rsFC between the aPFC and the inferior temporal gyrus, precentral gyrus, and postcentral gyrus, underscoring the implication of the aPFC in the occurrence of depression post-TBI.

In the context of non-TBI related depression, very few studies have examined rsFC of the aPFC specifically within the PFC. Examining the PFC broadly, increased rsFC between the PFC - specifically the inferior frontal gyrus—and amygdala was reported in patients with major depressive disorder (MDD) as compared to healthy controls, which decreased after treatment with antidepressant medication (41). The hyperconnectivity was interpreted as the PFC increasing its inhibition of the amygdala in depressed states, simultaneously leading to an increased frequency of negative emotions. Conversely, another study examined rsFC of the corticolimbic system to report reduced dlPFC-amygdala rsFC in unmedicated patients with MDD as compared to healthy controls (42). Moreover, recent seminal work has parceled brain activity/connectivity of patients with depression and anxiety disorders into 6 distinct biotypes in a study of task-based MRI (43). The profiles were based on symptom severity, behavioral performance, and response to treatment. Although the medial superior, anterior medial, and ventromedial prefrontal cortices emerged as key in differentiating the biotypes, this circuit-level approach precludes us from understanding the unique contributions of specific brain regions including the aPFC (44, 45). Therefore, targeted investigation into the aPFC is necessary to better understand its role in comorbid depression and TBI.

Given the lack of studies in this area, this present study aimed to contribute groundwork research towards the identification of a neural signature of depressive symptoms in chronic msTBI by examining rsFC of the aPFC to other brain regions voxel-wise. Thus, aPFC seed-based connectivity analysis comparisons were undertaken between the groups of patients with and without depression as well as healthy controls to examine group differences in bilateral aPFC rsFC. Based on the findings of rsFC alterations in the frontoparietal control network (46–48) and salience network (30, 49, 50), in depression and msTBI (22), and the occurrence of cognitive control deficits in both comorbidities (30, 32–34), we hypothesized reduced rsFC between the aPFC and other brain regions in the chronic msTBI groups as compared to healthy controls. Additionally, we also hypothesized reduced right aPFC rsFC with other regions in the group of patients with chronic msTBI compared to those with comorbid msTBI and depression (23).

## 2 Materials and methods

### 2.1 Participants

This is a secondary data analysis of prospectively collected data from the Toronto Rehab TBI Recovery Study, a longitudinal study examining neural, cognitive, and mental health recovery following msTBI (51–54). All patients provided written informed consent for their participation in the study.

#### 2.1.1 TBI groups*: msTBI-Dep* and *msTBI-Non-Dep*

Inclusion criteria for the parent study comprised: (1) diagnosis of msTBI; (2) between 18 and 80 years in age; (3) able to follow commands in English; (4) able to use at least 1 upper extremity; and (5) able to provide informed consent for participation or availability of a legal decision-maker. Exclusion criteria included: (1) prior history of TBI; (2) other pre-existing central nervous system disorder; (3) current diagnosis or history of a psychotic disorder; (4) persisting PTA at 6 weeks post-injury; and (5) metal implants that precluded MRI. Further details on the inclusion/exclusion criteria of the parent study are detailed in reports directly related to said study (51, 55). An additional inclusion criterion for the current study was availability of valid fMRI scans and a completed Personality Assessment Inventory (PAI) (56) questionnaire in the chronic stage of the msTBI (> 6 months post-injury). Pairwise deletions were made for patients with invalid responses on the PAI based on internal validity indices (56). Specifically, individuals with T scores ≥75, ≥ 73, ≥ 68, and ≥ 92 on the Infrequency, Inconsistency, Positive Impression Management, and/or Negative Impression Management (NIM-Tot) scale, respectively, were excluded (32). See Table 1.

**Table 1:** Demographic and injury characteristics*: msTBI-Dep* vs. msTBI-Non-Dep groups.

Demographics	Healthy controls Mean (SD)	*msTBI-Dep* Mean (SD)	*msTBI-Non-Dep* Mean (SD)	*p*-value	Effect size
Participants	17	13	19		
Age (years)	38.59 (15.12)	37.15 (13.69)	38.47 (16.71)	0.653	0.018
Sex (% males)	29.41%	53.85%	68.42%	0.067	0.336
YOE	15.12 (2.63)	14.15 (2.21)	15.42 (1.84)	0.315	0.050
Race					
White	-	11	16	1.00	0.161
Asian		2	2		
Other		0	1		
Time post-injury (months)	-	20.23 (8.53)	18.89 (10.58)	0.343	0.1737
History of depression (%)	-	15.38%	5.26%	0.737	0.220
LPTA					
1–7 days	-	4	3	0.198	0.338
1–4 weeks		4	11		
>4 weeks		5	3		
N/A		0	2		
Lowest GCS Score Reported	-	4.64 (1.61)	7.61 (3.58)	0.024*	0.3972
ACLOS (days)	-	38.27 (25.61)	34.89 (22.0)	0.808	0.0501
Mechanism of injury (%)					
MVA	-	69.23%	63.16%	1.00	0.214
Fall		30.77%	26.32%		
Assault		0%	5.26%		
Sports injury		0%	5.26%		
Other		0%	0%		
DEP-Tot	-	74.54 (13.61)	47.79 (5.96)	<0.001*	0.7907
DEP-C	-	69.00 (17.18)	47.89 (6.32)	<0.001*	0.6468
DEP-A	-	74.23 (13.39)	46.58 (6.73)	<0.001*	0.806
DEP-P	-	68.85 (12.15)	49.47 (6.75)	<0.001*	−2.011

SD, standard deviation; LPTA, length of posttraumatic amnesia; GCS, Glasgow Coma Scale; ACLOS, acute care length of stay; MVA, motor vehicle accidents; DEP-Tot, full scale total score from the Personality Assessment Inventory; DEP-C, Personality Assessment Inventory Depression-Cognitive subscale; DEP-A, Personality Assessment Inventory Depression-Affective subscale; DEP-P, Personality Assessment Inventory Depression-Physiological subscale; *Significant at *p* < 0.05.

Depression status was operationalized using a *T*-score cutoff of 60 on the PAI’s Depression clinical scale’s total score (DEP-Tot), as employed by other studies assessing depressive symptoms in TBI populations (57, 58). Participants with DEP-Tot *T-*scores ≥60 were assigned to the *msTBI-Dep group* (*n* = 13; mean = 74.54 (SD = 13.61)) and those with DEP-Tot scores <60 were placed in the *msTBI-Non-Dep group* (*n* = 19; mean = 47.79 (SD = 5.96)).

#### 2.1.2 Healthy control group

The *healthy control group* comprised *n* = 25 healthy controls matched on age and education to the msTBI groups. Inclusion criteria were: (1) between 18 and 80 years in age; (2) able to follow commands in English; and (3) commitment to completing the imaging scans. Exclusion criteria included: (1) previous history of TBI requiring hospitalization including concussion; (2) history of any disease affecting the central nervous system; (3) current diagnosis of depression; and (4) presence of magnetic materials affecting MRI scan acquisition. Applying these criteria, we obtained a sample of *n* = 17 healthy controls.

### 2.2 Materials

#### 2.2.1 Personality assessment inventory (PAI)

The PAI is a 344-item self-report scale, with high internal consistency (56) and construct validity (59). The PAI has been validated for msTBI (54). To classify patients into study groups, we employed the total score of the Depression clinical scale (DEP-Tot), which represents a composite of its three subscales: Depression-Cognitive (DEP-Cog), Depression-Affective (DEP-Aff), and Depression-Physiological (DEP-Phys) symptoms.

#### 2.2.2 Neuropsychological tests

We examined the performances of the *msTBI-Dep* and *msTBI-Non-Dep* groups on a range of neuropsychological tests to identify potential differences between the groups that might contribute to aPFC rsFC differences. A comprehensive clinical battery measuring attention, speed of processing, verbal and visuospatial memory, executive functioning, verbal and performance IQ, and estimated premorbid IQ was administered by a trained psychometrist under the supervision of a neuropsychologist. Additional details of the battery are listed in a previous publication by our group (52).

### 2.3 Design and procedures

#### 2.3.1 Imaging data acquisition

MRI data were obtained using a General Electric (GE) Signa-Echospeed 1.5 Tesla HD scanner (SIGNA EXCITE, GE Healthcare, Milwaukee, Wisconsin) with an 8-channel head coil configuration. First, high-resolution T1-weighted three-dimensional gradient-echo echo-planar images were acquired with the following parameters: sagittal T1-weighted spin echo, repetition time = 300 msec, echo time = 13 msec, slice thickness = 5 mm no gap, slice spacing of 2.5 mm, matrix = 256 × 128, and field of view (FOV) = 22 cm. Resting-state scans (rs-fMRI) lasting 5 min were then acquired via whole-brain gradient-echo echoplanar images with the following parameters: repetition time = 2,000 msec, echo time = 40 msec, flip angle = 85°, slice thickness = 5.0 mm and 150 axial slices, interleaved in order. Participants were instructed to keep their eyes closed for the 5-min resting scans.

#### 2.3.2 MRI data preprocessing

Resting-state fMRI data were preprocessed using FMRIB Software Library (FSL version 5.0.1; http://fsl.fmrib.ox.ac.uk/) FEAT. This was done using similar steps to those performed in a previous study by our lab group (55). Preprocessing included: (1) discarding the first five functional volumes, resulting in 145 volumes for analysis; (2) skull stripping using FSL BET; (3) motion correction using FSL MCFLIRT; (4) spatial smoothing with a Gaussian kernel of 5 mm full-width at half-maximum; (5) Gaussian-weighted high pass temporal filter (100 s); (6) artifact denoising via ICA-AROMA (60) which has been validated for seed-based analyses (61); (7) cerebrospinal fluid and white matter signal regression with thresholds of 1 and 0.98, respectively, via FSL FAST; and (8) linear registration with 12 degrees of freedom to MNI152 through FSL FLIRT. The final outputs were in Montreal Neurological Institute (MNI) space (62). Global signal regression was not applied given that it can introduce anticorrelations into the functional data (60, 63). For quality assurance, the data were visually inspected by a neuroradiologist following registration to ensure that there were no contusions and/or excessive motion (*n* = 3 patients were excluded accordingly).

#### 2.3.3 Whole-brain seed-based connectivity analysis

Following preprocessing, whole-brain seed-based connectivity analyses were implemented through FSL FEAT (64). Two 5 mm radius spheres were created and centered on the following MNI coordinates: left aPFC: −36, 57, 9; right aPFC: 34, 52, 10. These ROI were delineated following the approach of a previous study identifying significant aPFC rsFC differences in patients with msTBI in comparison to healthy controls (22). The average BOLD signal time series within each aPFC seed were extracted and entered as the primary regressors in a first-level general linear model (GLM) for the left and right aPFC, independently. In all first-level analyses, cluster-based thresholding was employed with a z-threshold of 3.1 and a significance level of 0.05. FSL FILM pre-whitening was applied to correct for time series autocorrelations and improve estimation accuracy.

#### 2.3.4 aPFC rsFC group comparisons

To identify bilateral aPFC rsFC differences across the *msTBI-Dep*, *msTBI-Non-Dep*, and *healthy control* groups, higher-level group comparisons were conducted via an *F*-test in FSL (65). The *F*-test simultaneously assessed all possible pairwise differences between the three groups, controlling for age and education. For any significant comparisons that emerged (FWE-corrected *p* < 0.05), two-tailed unpaired *t*-tests were subsequently performed to investigate the directionality of findings.

To preserve the study’s statistical power, we undertook a data-driven approach in addition to consulting the literature to identify the covariates that were included, and thus, controlled for in the GLM of the seed-based analyses. First, education was included due to the high variability in education attained across participants (range: 9 to 18 years) as well as the differences between the *msTBI-Dep, msTBI-Non-Dep, and healthy control* groups in education with a moderate effect size (*H*(2, *N* = 49) = 2.31, *p* = 0.315, *ε^2^* = 0.0.050). Second, it was important to control for age given its documented effects on rsFC of various brain regions and networks in depression (66, 67). Apart from its effect on rsFC, older age at injury also portends poorer functional outcomes post-TBI (53, 68). Despite the wide range in inclusion criteria for age, only 2 patients were 65+, distributed across the *healthy control* and *msTBI-Dep* groups. Altogether, age and education (both measured in years) were centered over the global mean of each group and added as covariates of non-interest. Total intracranial volume (TIV) was not added as a covariate because of the limited sample size. Despite this, all fMRI scans were normalized and registered to the standard MNI-152 template. This would’ve at least partially reduced potential influences of TIV (69).

Between-group differences in aPFC rsFC were assessed using FSL’s non-parametric permutation-based Randomize. Threshold-free cluster enhancement (TFCE) was used with 5,000 permutations to avoid the use of an arbitrary cluster-forming threshold, and family-wise error (FWE) correction was undertaken to correct for multiple comparisons. TFCE is preferred over traditional cluster thresholding methods given that it substantially reduces the rate of false positive results (70, 71). Significant clusters were localized and labeled using FSL’s Harvard-Oxford Cortical and Subcortical Structural Atlas (72). The sizes of these clusters were identified and denoted k. Effect sizes for the connectivity maps, that is, Cohen’s *d* values, were computed from *t*-statistic maps using the standard Cohen’s d calculation formula for two-sample *t-*tests.

#### 2.3.5 Statistical analyses

Statistical analyses for all demographic, clinical, and neuropsychological assessment data were carried out on IBM SPSS Statistics (version 28.0.1.0). The data were first subjected to normality testing using the Shapiro–Wilk normality test. Subsequently, the *msTBI-Dep group* and the *msTBI-Non-Dep group* were compared on all demographic, clinical and neuropsychological variables to identify any significant group differences that could influence rsFC findings. An *α* level of 0.05 was utilized to identify significant differences. As for effect sizes, Cohen’s *d* was computed for independent-samples *t*-tests, effect size *r* was computed for Mann–Whitney U tests, KW-epsilon squared for the Kruskal-Wallis test, and Cramer’s *V* for Fisher’s exact test (see Table 1).

## 3 Results

### 3.1 Demographic and injury-related variables

The *msTBI-Dep* and *msTBI-Non-Dep* group differed in terms of the lowest GCS recorded, which was significantly higher in the *msTBI-Non-Dep group* with a medium effect size (*U* = 49.5, *p* = 0.024, *d* = 0.3972). Length of post-traumatic amnesia and acute care length of stay did not differ significantly between the two msTBI groups (*p*’s > 0.05). Otherwise, there were no significant differences between the three study groups in the remaining demographic, injury-related, and clinical variables.

### 3.2 Neuropsychological tests

With regards to the neuropsychological assessments examined, no significant difference was found between the *msTBI-Dep group* and the *msTBI-Non-Dep group* on any assessment, and the effect sizes were all small with the exception of the Stroop color-naming, word-reading, and Stroop interference tests which were medium (Cohen’s *d* = 0.4–0.6).

### 3.3 aPFC rsFC group comparisons

**Figure 1:**
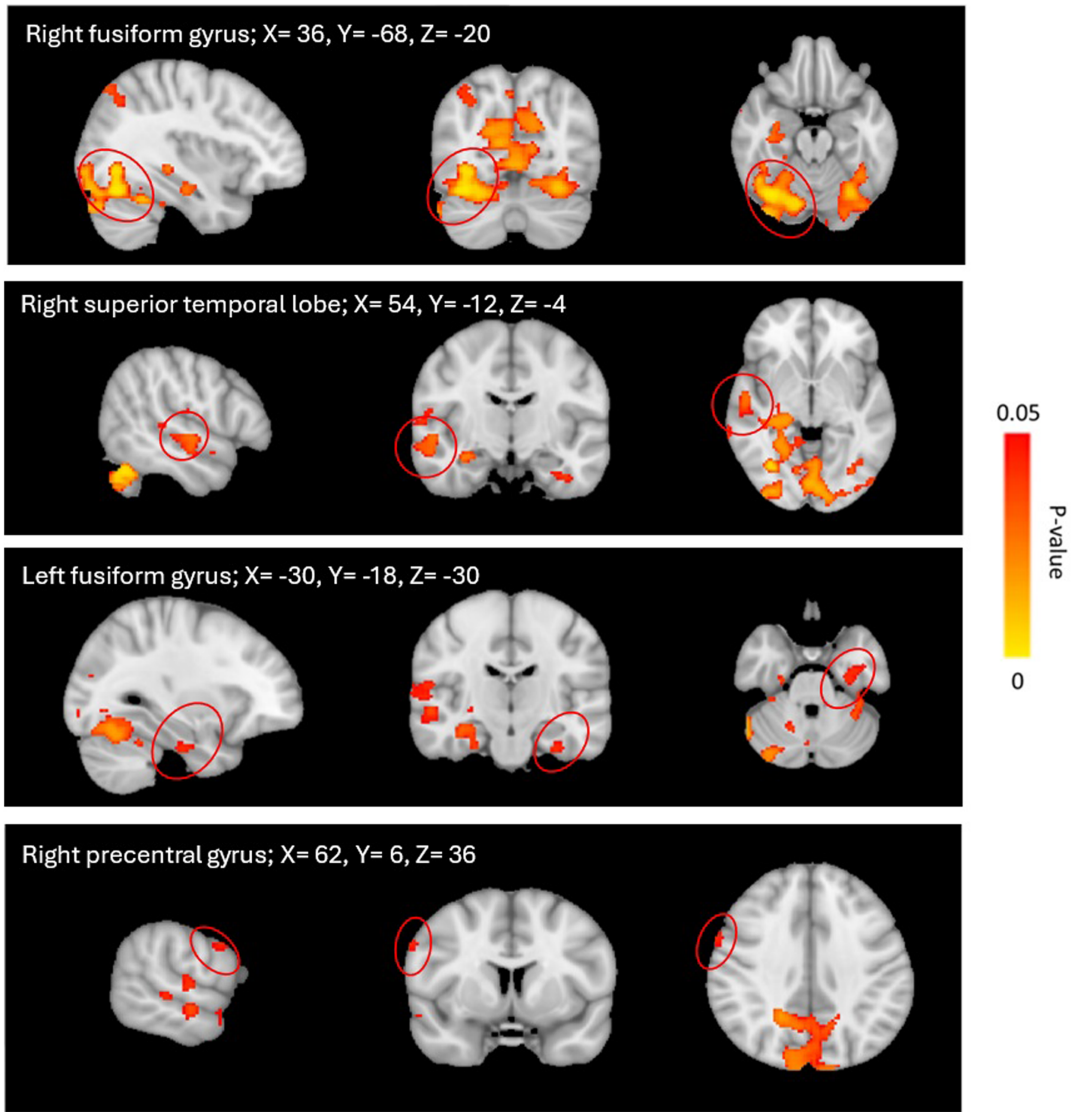
LaPFC rsFC Comparisons: *msTBI-Dep Group > healthy control group.* Significant clusters obtained from the *F*-test computed for the left anterior prefrontal cortex, specifically the *msTBI-dep* > *healthy control* contrast, after controlling for age and education. Images represent threshold-free cluster enhancement and family-wise error rate-corrected *p*-values thresholded at an *α* level of 0.05, overlaid on a standardized MNI-152 brain. The left hemisphere of the brain corresponds to the right side in this image and vice versa. See Table 2.

There were no significant differences between the *msTBI-Non-Dep group* and the *msTBI-Dep group* nor the *msTBI-Non-Dep* group and the *healthy control group* (FWE-corrected *p* < 0.5) in bilateral aPFC rsFC. However, rsFC of the left aPFC with 4 brain regions was significantly higher in the *msTBI-Dep group* as compared to the *healthy control group*, with large effect sizes. The 4 regions consisted of the left fusiform gyrus (BA37; *t* = 3.65, TFCE = 22,729, FWE-corrected *p* = 0.041, *k* = 119, Cohen’s *d* = 1.35), the right fusiform gyrus (BA37; *t* = 4.39, TFCE = 30,761, FWE-corrected *p* = 0.01, *k* = 11,867, Cohen’s *d* = 1.62), the right superior temporal lobe (STL; BA22; *t* = 4.43, TFCE = 24,941, FWE-corrected *p* = 0.032, *k* = 685; Cohen’s *d* = 1.63), and the right precentral gyrus (BA4; *t* = 4.34, TFCE = 22,443, FWE-corrected *p* = 0.042, *k* = 29, Cohen’s *d* = 1.60). These differences were non-significant between the two other study groups (*p* > 0.05). See Table 2 and Figure 1.

**Table 2:** Left *aPFC rsFC* group comparisons*: msTBI-Dep Group > healthy control group.*

Region	Peak Voxel Coordinates^a^			Cluster Size (*k*)	TFCE	*p*-value^b^	Effect Size^c^
	X	Y	Z				
Fusiform gyrus	36	−68	−20	11,867	30,761	0.013*	1.61
Superior temporal lobe	54	−12	−4	685	24,941	0.032*	1.63
Fusiform gyrus	−30	−18	−30	119	22,729	0.041*	1.35
Precentral gyrus	62	6	36	29	22,443	0.042*	1.60

Regions showing significant differences in left aPFC rsFC between the msTBI-Dep group and the healthy control group. aPFC, anterior prefrontal cortex; rsFC, resting-state functional connectivity; msTBI, moderate-to-severe traumatic brain injury; hem, hemisphere; TFCE, threshold-free cluster enhancement.

a Cluster locations reported in MNI coordinates.

b Analyses were conducted using a conservative FWE-corrected threshold of 0.05, through 5,000 permutations.

c Effect size computed via Cohen’s d and T-statistics.

## 4 Discussion

The overarching aim of this study was to address a gap in the research with regard to the clinical neuroimaging biomarkers of depression in msTBI (21). This was the first study to examine the question of biomarkers of depression in bilateral aPFC rsFC through seed-based connectivity analyses in (1) a homogenous group of patients with msTBI (i.e., not integrated with mild TBI) and (2) in the chronic stages of injury.

We found increased rsFC in the *msTBI-Dep group* as compared to the *healthy control group* between the left aPFC and four sensory and motor regions (bilateral fusiform gyri, right STL, and right precentral gyrus). In this preliminary study, the implication of the aPFC - a brain region involved in cognitive control and salience detection - and these 4 regions aligns with past literature reporting rsFC alterations to these aforementioned modalities in both msTBI (73) and depression (32, 74) populations.

Broadly, dysfunction of the PFC has been consistently implicated in affective disorders, including MDD (75). Focusing on the aPFC specifically, it is associated with the salience network, and thus, implicated in salience detection, attention control, and emotional regulation (39, 76). Additionally, as a key hub within the frontoparietal control network, it contributes to cognitive control (36) and action selection through reward tracking (77). Dysfunction of the salience and frontoparietal control networks are commonly reported in clinically depressed populations (30, 78, 79). Despite this, there exists a very limited range of studies examining aPFC rsFC in depression following chronic msTBI (22, 23). It is therefore important to interpret these findings in the context of the cognitive and affective roles that these implicated regions have.

As it relates to brain-behavior relationships, the fusiform gyrus has been known to facilitate object and facial recognition (80) and plays pivotal roles in affect and emotional processing (81–83). Similarly, in addition to coordinating motor movements as part of the primary motor cortex (84, 85), the precentral gyrus plays a critical role in regulating emotional circuitry involving the amygdala (86, 87). Increased activity of the precentral gyrus was reported during the perception of threatening emotional stimuli as compared to neutral stimuli, supporting the engagement of this motor region in emotional processing (88). Not surprisingly, increased rsFC between motor regions including the supplementary motor area and self-related regions including the pregenual ACC was reported in patients with depression as compared to healthy controls (89). Lastly, as part of the affective network (90), the STL has been implicated with emotional processing in patients with MDD (91, 92) as well as healthy patients (93). Evidently, regardless of whether it is a primary function or not, these sensory and motor regions have documented roles in emotional processing and regulation.

Frontal regions, including the PFC, exert top-down control over emotion processing regions as a regulatory mechanism (94–96). In patients with mild TBI, Iraji et al. (97) reported increased rsFC between the aPFC and the thalamus at the acute stage post-injury. This hyperconnectivity was hypothesized to serve as a compensatory mechanism for depression through capitalizing on additional networks and connectivity. Therefore, given the roles of these four regions, it is possible that the increased rsFC between the aPFC and these sensory and motor regions reflects an attempt to increase top-down control over the excessive bottom-up emotional processing experienced in depression in the *msTBI-Dep group*. In other words, this hyperconnectivity could be a marker of compensatory mechanisms that down-regulate the heightened reactivity to negative emotional stimuli that emerges in depression following injury (97). However, such hyperconnectivity is ultimately without functional gain as reflected by the elevated PAI DEP-Tot scores, and hence, the presence of depressive symptoms in the *msTBI-Dep group*.

Conversely, the observed pattern of non-adaptive - or potentially maladaptive - hyperconnectivity between the aPFC and the four sensory and motor regions could itself be an underlying neuropathology of depressive symptomatology in the *msTBI-Dep group*. This hypothesis is supported by the literature findings wherein patients with MDD exhibited PFC-amygdala hyperconnectivity that decreased over the course of 8 weeks of treatment with antidepressant medication (41). Interestingly, hyperconnectivity within higher-order cognitive and sensory networks was also reported in chronic msTBI, which the authors hypothesized to be attributable to compensatory mechanisms and/or underlying TBI symptomatology (98). Because the msTBI groups had non-significant aPFC rsFC differences in the present study, it is thus plausible to consider that the aPFC hyperconnectivity in our *msTBI-Dep group* may be due to either compensatory mechanisms, underlying TBI symptomatology, or both. Although future research is needed to delineate the mechanisms behind these rsFC changes, the findings of this present study show that aPFC rsFC may have potential as a neuroimaging marker for characterizing the changes that occur in the presence of depressive symptoms in patients with chronic msTBI.

### 4.1 Clinical implications

Despite being one of the most common sequelae of msTBI, current clinical treatment strategies for the management of depression post-TBI are lacking (10, 21). This renders chronic msTBI patients at an increased risk of developing depression and its associated functional impediments (99, 100). What is more, associations between rsFC and depression scores have been reported in chronic msTBI in the absence of associations between whole-brain grey matter volume and depression scores, contributing to the psychiatric sequelae that emerge after TBI while evading detection through structural imaging tools (14).

The observed aPFC hyperconnectivity in depression post-TBI informs treatment research through aiding the prediction and monitoring response to treatment. Consequently, non-invasive neuromodulation-based therapies, e.g., transcranial magnetic and deep brain stimulation can be utilized to normalize aberrant aPFC rsFC, alleviating depressive symptoms and augmenting quality of life (11–13). This is particularly important given previous research from our lab showing an escalation in the number of patients with clinically significant symptoms of depression in the chronic stages of msTBI (101).

## 5 Strengths, limitations, and future directions

In terms of strengths, employing seed-based connectivity analyses to identify a signal for depressive symptoms post-TBI signifies that our findings are restricted to a specific seed region as opposed to the network(s) with which the region is associated. Although network-based approaches such as independent component analysis enable a more holistic understanding of brain rsFC, the aPFC seed-based connectivity analyses undertaken in this study serve as a starting point for understanding brain rsFC alterations in a research area that is yet to be explored.

Small sample size was a notable limitation of this study, which may have limited our statistical power; it also precluded control for additional potential confounds within the models such as TIV, injury severity, history of depression or anxiety, and participant sex which may have affected the aPFC rsFC reported. However, although participant sex has been reported to impact rsFC in depression (102, 103), there were no significant differences in sex distribution between our three study groups via Fisher’s exact test (*p* > 0.05, Cramer’s *V* = 0.34). Given that the effect size was moderate, additional sensitivity analyses were run to complement this finding. This included correlational analyses between sex distribution and the PAI DEP-Tot depression scores which were also non-significant in our msTBI groups (*p* = 0.615). Similarly, although the inclusion criteria for age were 18–80, only 2 patients were 65+, with this variable controlled for within the GLM.

Because this study was a secondary analysis on prospectively collected data, we did not have control over the study parameters and outcomes, including the imaging scanner acquisition hardware and software. One notable limitation in this regard was that the neuroimaging data employed in this study were acquired with a 1.5 Tesla scanner, which may have limited our signal-to-noise ratio, and thus, spatial resolution.

Replication studies should address these limitations. To build additional mechanistic insight, future studies should consider exploring aPFC rsFC using a seed-to-region of interest (ROI) analysis inclusive of the bilateral aPFC seeds and the precentral gyrus, the fusiform gyrus, and the STL as ROI. Future analyses could also be expanded to include ROIs previously implicated in depression following chronic msTBI (14, 24) as well as ROIs implicated in non-TBI related depression (104–107). The latter is warranted in light of the reported similarities in neurophysiological responses to depression occurring within and outside the context of msTBI (14, 108, 109). Such analyses would enrich our understanding of rsFC alterations occurring between the left aPFC and sensory and motor regions following chronic msTBI and whether the observed hyperconnectivity constitutes vulnerability or a compensatory reaction to depression following chronic msTBI.

## 6 Conclusion

This present study, to our knowledge, is first to investigate aPFC rsFC in a group of patients with exclusively msTBI (i.e., without patients in the mild range of TBI) and in the chronic stages of msTBI. Our findings revealed that aPFC-sensory/motor rsFC was significantly increased in the *msTBI-Dep group* compared to the *healthy control group*, though the nature of this hyperconnectivity necessitates further investigation. Our results provide support to findings established in depression literature, in the context of comorbid depression and msTBI. Altogether, these preliminary findings contribute novel empirical data towards characterizing the functional basis of depression in chronic msTBI.

## Data Availability

The raw data supporting the conclusions of this article will be made available by the authors, without undue reservation.
